# Gene-Environment Interactions in Progressive Supranuclear Palsy

**DOI:** 10.3389/fneur.2021.664796

**Published:** 2021-04-09

**Authors:** Irene Litvan, James A. Proudfoot, Eden R. Martin, David Standaert, David Riley, Deborah Hall, Connie Marras, Ece Bayram, Richard M. Dubinsky, Yvette Bordelon, Stephen Reich, David Shprecher, Benzi Kluger, Christopher Cunningham, Gerard D. Schellenberg, Joseph Jankovic

**Affiliations:** ^1^Department of Neurosciences, Parkinson and Other Movement Disorders Center, University of California, San Diego, La Jolla, CA, United States; ^2^Clinical and Translational Research Institute, University of California, San Diego, La Jolla, CA, United States; ^3^John P. Hussman Institute for Human Genomics, Miller School of Medicine, University of Miami, Miami, FL, United States; ^4^Department of Neurology, University of Alabama at Birmingham, Birmingham, AL, United States; ^5^InMotion, Warrensville Heights, OH, United States; ^6^Department of Neurological Sciences, Rush University, Chicago, IL, United States; ^7^Morto and Gloria Shulman Movement Disorders Centre and the Edmond J. Safra Program in Parkinson's Research, Toronto Western Hospital, University of Toronto, Toronto, ON, Canada; ^8^Department of General Neurology, University of Kansas Medical Center, Kansas City, KS, United States; ^9^Department of Neurology, University of California, Los Angeles, Los Angeles, CA, United States; ^10^Department of Neurology, University of Maryland School of Medicine, Baltimore, MD, United States; ^11^Banner Sun Health Research Institute, Sun City, AZ, United States; ^12^Department of Neurology, University of Utah, Salt City, UT, United States; ^13^Department of Neurology, University of Colorado, Denver, CO, United States; ^14^Division of Movement Disorders, Department of Neurology, University of Louisville School of Medicine, Louisville, KY, United States; ^15^Department of Pathology and Laboratory Medicine, University of Pennsylvania Perelman School of Medicine, Philadelphia, PA, United States; ^16^Parkinson's Disease Center and Movement Disorders Clinic, Department of Neurology, Baylor College of Medicine, Houston, TX, United States

**Keywords:** progressive supranuclear palsy, gene, environment, epidemiology, risk factors

## Abstract

Several genetic and environmental factors have been reported in progressive supranuclear palsy (PSP), although none were identified as a definitive cause. We aimed to explore potential gene-environment interactions in PSP. Two hundred and ninety two PSP cases and 292 controls matched for age, sex, and race from the ENGENE-PSP were analyzed to determine the association between PSP and minor alleles of 5 single nucleotide polymorphisms (SNPs) in 4 genes (MAPT, MOBP, EIF2AK3, and STX6), which were previously associated with PSP risk. Interactions between these SNPs and environmental factors, including previously reported occupational and agricultural risk factors for PSP, were assessed for PSP odds and age of symptom onset. Minor alleles of MAPTrs242557 and EIF2AK3rs7571971 were individually associated with increased odds; MAPTrs8070723 minor alleles were associated with lower PSP odds. There were several gene-environment interactions for PSP odds and age of symptom onset, however, they did not remain significant after FDR-correction. Larger scale studies are required to determine potential interactions.

## Introduction

Progressive Supranuclear Palsy (PSP) is the second most common cause of neurodegenerative parkinsonism after Parkinson's disease (1). First described in 1964, classic PSP is characterized by early postural instability, frontal cognitive disturbances, pseudobulbar palsy, and vertical supranuclear gaze palsy, preceded by slowing of vertical saccades (2). PSP results in rapid deterioration of quality of life, with median survival time estimated at 7–8 years (3, 4). Previous estimated prevalence was 5–6 cases per 100,000 (5), but recent studies show that true PSP prevalence is higher. Recent age-adjusted prevalence estimates in Europe are 8.8–10.8 cases per 100,000 (6, 7), and in Yonogo Japan, age-adjusted PSP prevalence increased from 5.8 cases per 100,000 in 1999 (8) to 17 cases per 100,000 in 2010 (9).

Few studies have investigated the possible genetic and environmental causes of PSP. Lower levels of education (10–12), drinking well-water, prior use of firearms associated with higher blood lead levels (13), and exposure to industrial metals (14) have been reported as environmental risk factors; whereas genetic risk factors include variants of MAPT, MOBP, EIF2AK3, and STX6 (15–18). None of these risk factors have been identified as a definitive cause of PSP, and PSP is more than likely caused by a complex interaction of genetic predisposition and environmental risk factors. However, there are no prior studies investigating gene-environment interactions for PSP risk. To address this gap, we designed a large, multi-center case-control study, Environmental-Genetic PSP (ENGENE-PSP), to begin exploring the interactions of genetic and environmental factors in PSP. We also assessed whether the gene-environment interactions impact age of symptom onset in PSP. Our previous report in this cohort showed that higher PSP odds were associated with more years of drinking well-water and not having a college degree (10). As our prior analysis focused specifically on environmental and occupational risk factors in the same cohort, we did not repeat the analysis for the environmental factors, but rather focused on genetic factors and gene-environment interactions.

## Materials and Methods

### Study Population

Cases and controls were recruited from 15 sites throughout North America between October 1, 2006 and February 1, 2013, as previously reported (10). Briefly, the sampling framework for the study included the catchment area surrounding each participating clinical center, and referrals from outside these areas were sent to their nearest participating site. Cases were confirmed to have PSP by the Principal Investigator of each participating site. To be included in the study, cases had to be diagnosed on-site within the past year and meet the NINDS-PSP Diagnostic Criteria for Clinically Probable or Clinically Possible PSP (2). Exclusion criteria included other central nervous system pathology, severe speech and cognitive impairment that could have interfered with recall of life events. Ninety-three percentage of cases had a Mini Mental State Examination score greater than 24. Majority of the PSP cases had PSP-Richardson's syndrome (*n* = 265, 90.8%) and there was a minority with PSP-parkinsonism (*n* = 17, 5.8%) (19).

**Figure 1:**
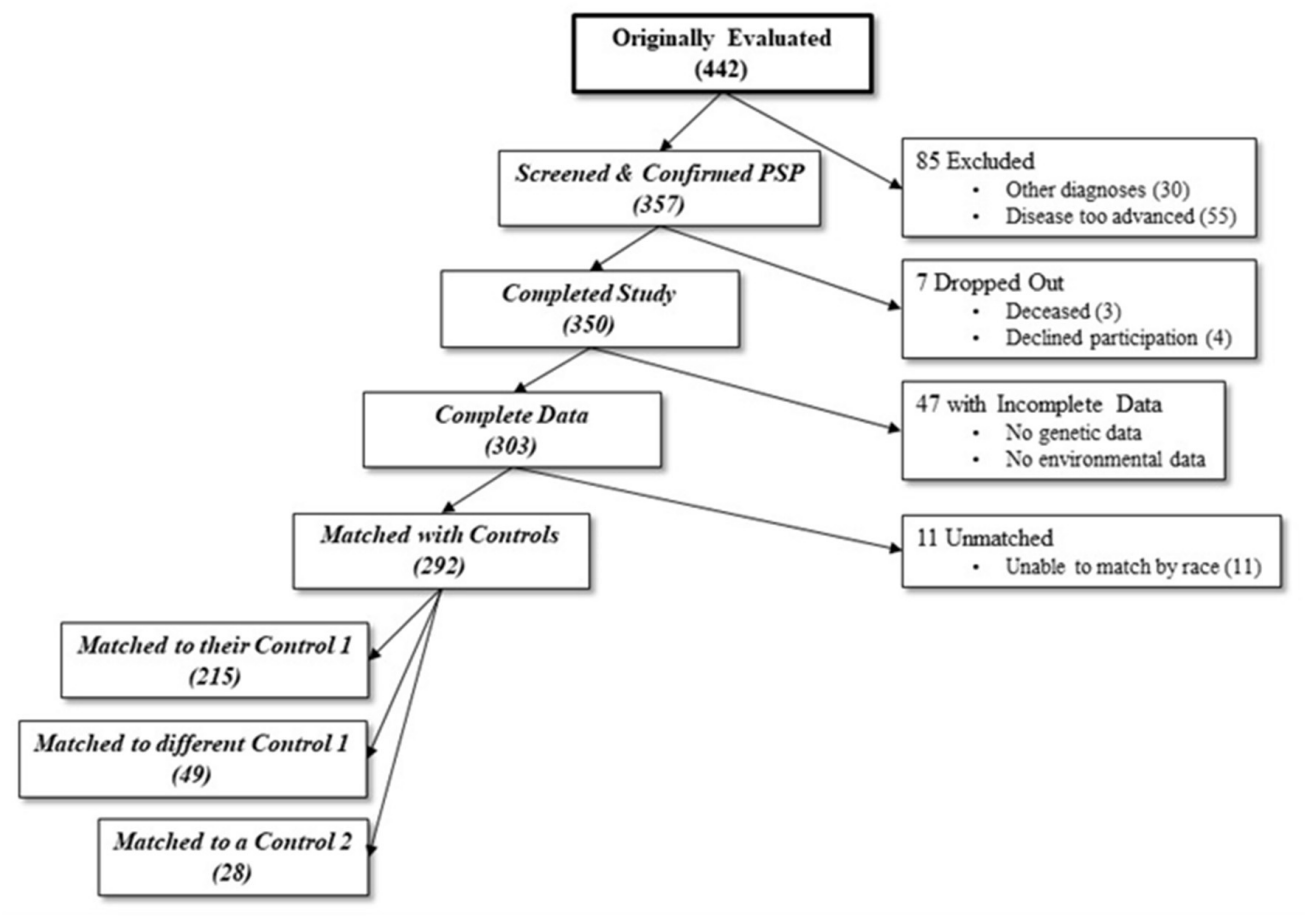
Number of participants at each stage of the study. From an initial sample of 442, 139 were excluded due to either incompatible diagnoses, too advanced disease, dropping out, or incomplete data. An additional 11 were excluded due to inability to match for race with a control. 292 final cases were matched with either their original control 1 (non-blood relative), a different case's control 1, or a different case's control 2 (spouse or caretaker).

Each subject was recruited together with two controls: an age (±10 years) and sex-matched non-blood relative (Control 1), and the subject's spouse or primary care partner (Control 2). To increase the sample size of this study we included both control 1 and control 2. Controls were age, race and sex-matched subjects, and although in-person examinations were not performed similar to the examination of cases, all controls screened negative for both parkinsonism and dementia using the Telephone Interview of Cognitive Status and Telephone Questionnaire for Parkinson's disease, respectively (20). Most cases were preferentially matched with their own recruited Control 1 (73.6%), with the remainder of cases being matched with either a different Control 1 (16.8%) or Control 2 (9.6%) (Figure 1). From an initial cohort of 350 PSP subjects (10), the analyses included 292 matched with 292 controls by race, sex, and age (±10 years, average 2.8 ± 2.5 years). Fifty-eight of the 350 cases were excluded from the analyses because of incomplete data (*n* = 47) or different race than the age and sex matched control (*n* = 11). The excluded cases were similar to the included cohort in age (68.76 ± 7.01, *P* = 0.125) (Table 1). However, compared to the included cases, the excluded cases had an almost-significantly larger proportion of men and were significantly different in terms of race, education level, and disease duration.

**Table 1:** Mean (standard deviation) and count (%) for demographic variables of included and excluded PSP cases.

	**Excluded (*n* = 58)**	**Included (*n* = 292)**	***p*-value**
**Age**	67.38 (7.06)	69.03 (6.98)	0.125
**Sex**			0.063
Female	21 (36.2%)	145 (49.7%)	
Male	37 (63.8%)	147 (50.3%)	
**Race**			<0.001***
Asian or Pacific Islander	5 (8.6%)	8 (2.7%)	
Black or African-American	5 (8.6%)	1 (0.3%)	
Latina/Latino or Hispanic	4 (6.9%)	3 (1.0%)	
Native American or Indigenous	2 (3.4%)	0 (0.0%)	
White or European-American	42 (72.4%)	280 (95.9%)	
**Highest Degree**			0.022*
Grade school (grades 1–8)	4 (6.9%)	2 (0.7%)	
High school (grades 9–12)	5 (8.6%)	41 (14.0%)	
High school diploma	19 (32.8%)	85 (29.1%)	
College diploma	13 (22.4%)	89 (30.5%)	
Technical or trade school diploma	1 (1.7%)	1 (0.3%)	
Graduate school diploma	16 (27.6%)	74 (25.3%)	
**Disease Duration**	3.20 (1.61)	4.22 (2.24)	<0.001***

*Significance is determined by the Mann-Whitney U test for continuous variables and Fisher's exact test for categorical variables*.

Statistical significance is marked with ^*^ for P < 0.05, ^****^ for P < 0.001.

Age of symptom onset was calculated from the first symptom (motor or non-motor PSP symptom) recalled by the patient and family. For controls, we used a “reference date” defined as the age of symptom onset of the matched case. PSP phenotypes of the cases were determined based on the Movement Disorders Society PSP clinical diagnostic criteria (19). This study was approved by the Institutional Review Board of each institution (IRB #111729), and each participant signed a written informed consent.

### Genetic Analyses

PCR using TaqMan genotyping assays (Thermo Fisher) was used to determine expression of five single-nucleotide polymorphisms (SNPs) previously associated with PSP (17). Predesigned assays included C__1016016_1 for rs242557 (MAPT), C_29297996_10 for rs8070723 (MAPT), C__75367_10 for rs1768208 (MOBP), and C__20893_10 for rs7571971 (EIF2AK3). A custom assay (design ID AHGJ5AO, rs1411478) was used to genotype STX6, with the forward primer GGTAGGCAAAAGGTGCTATGGA, reverse primer GTCCCAGCACCCTGTCAA, reporter 1 sequence CCCAGAGAAGAAGAC, and reporter 2 sequence CCAGAGGAGAAGAC. Genotypes were recorded for each SNP and compiled into a database for further analysis. Genetic information was encoded for each genotype in terms of number of minor alleles counts per subject, with homozygous major alleles encoded as 0, heterozygous as 1, and homozygous minor alleles as 2 (Table 2). For example, for MAPT rs242557 A is the minor allele, thus, each subject A/A is encoded as 2, A/G as 1, and G/G 0.

**Table 2:** Minor allele frequency by participant groups.

**Single nucleotide polymorphism**	**Gene**	**Minor allele**	**Minor allele frequency-Case**	**Minor allele frequency-Control**
MAPT	rs242557	A	0.536	0.312
MAPT	rs8070723	G	0.050	0.248
MOBP	rs1768208	T	0.344	0.332
EIF2AK3	rs7571971	T	0.307	0.250
STX6	rs1411478	A	0.455	0.416

### Environmental Factors

To assess occupational exposures to toxic substances, all participants were given a telephone occupational questionnaire modified from Stewart and colleagues (21), in which they listed all jobs held for over 6 months between the ages of 16 and 10 years before their PSP symptom onset (or reference date for controls). The methodology was previously reported in detail (10). Briefly, participants listed their company name, job section, position held, number of years in their position, duties, tools and equipment used, and possible chemical exposures. They also self-reported environmental exposures to various substances, including organic solvents, pesticides, herbicides, fungicides, and other chemicals. Due to usual inaccuracies with self-reported work-related toxic exposures (under or over-reporting exposures), we selected a priori a more objective assessment by an independent team consisting of a toxicologist and an industrial hygienist to review the reported lifetime occupational data and assign their own estimates of occupational exposures to chemicals in general, metals, pesticides, and organic solvents, blinded to case/control status (10). Due to the self-reported nature of the data, it wasn't possible to accurately quantify toxic exposure; exposures were instead listed as a binary yes/no values. Levels of exposure were defined as “high” for directly working with the chemical or “low” for exposure via a proximate worker working with the chemical (i.e., manager).

Other recorded environmental exposures included residential history, well-water intake, history of having lived one mile of an agricultural area, hobbies, family history of neurological disorders, military history and exposures, and specifics regarding gardening and lawn care (10).

### Statistical Analyses

All analyses were done with the R statistical programming language (version 3.6.1). We fitted conditional logistic regression models to account for the matched structure of the data using case/control status as an outcome. Two regressions were fitted; univariate models for each genetic factor, and a model which included the interactions for each genetic and environmental factor. For age at symptom onset, we used a simple linear regression and fitted two models in a similar fashion as the conditional logistic regression analysis. Model diagnostics (residual plots, qq-plots) were performed to ensure that the assumptions of the linear models were met by the data. All models were re-ran to include age, race, and sex as covariates; and the results did not change. A *p*-value of less than 0.05 was suggestive of significance for this study in view of evaluating the previously 5 significant SNPs. A false discovery rate (FDR) *p*-value correction separately for outcomes was also performed to maintain a type I error rate of 5%. Power calculation was based on detecting a significant variation in a continuous factor across genetic/case groups. With a total sample size of 584, we had an 80% power to detect an effect size (Cohen's F) of 0.02, which has previously been described as small (22).

## Results

### Demographics

Cases and controls were similar in age, sex, and race, reflecting the matched case-control design of this study. Overall, mean age was 69.01 years, 50.3% of the participants were male, and 95.9% were white or European-American (Table 3). Majority of the participants received a college/trade school degree or higher, and controls had higher degrees than cases (*P* < 0.001). Mean (standard deviation) disease duration was 4.22 (2.24) years for PSP cases.

**Table 3:** Demographics by case status.

	**Case (*n* = 292)**	**Control (*n* = 292)**
**Age**	69.03 (6.98)	68.98 (7.46)
**Sex**		
Female	145 (49.7%)	145 (49.7%)
Male	147 (50.3%)	147 (50.3%)
**Ethnic Group**		
Asian or Pacific Islander	8 (2.7%)	8 (2.7%)
Black or African-American	1 (0.3%)	1 (0.3%)
Latina/Latino or Hispanic	3 (1.0%)	3 (1.0%)
White or European-American	280 (95.9%)	280 (95.9%)
**Highest Degree**		
Grade School	2 (0.7%)	5 (1.7%)
High School	126 (43.2%)	85 (29.1%)
College / Trade School	90 (30.8%)	84 (28.8%)
Graduate School	74 (25.3%)	118 (40.4%)

*Data is presented as mean (standard deviation) and count (percentage) for continuous and categorical variables, respectively*.

### Genetic Associations With PSP

In univariate analysis, the minor allele of MAPTrs242557 (A) (a marker tagging the H1c sub-haplotype) and the minor allele of EIF2AK3rs7571971 (T) were significantly associated with increased odds for PSP, while the minor allele of MAPTrs8070723 (G) was associated with lower odds for PSP (Table 4).

**Table 4:** Genetic factors associated with PSP risk.

	**Odds ratio**	**95% Confidence Interval**	***p*-value**
**Genetic factors**			
MAPTrs242557	2.315	(1.794, 2.988)	8.533e-13***
MAPTrs8070723	0.152	(0.091, 0.253)	5.05e-13***
MOBPrs1768208	1.057	(0.839, 1.331)	0.639
EIF2AK3rs7571971	1.340	(1.028, 1.748)	0.031*
STX6rs1411478	1.183	(0.932, 1.502)	0.167

*Odds ratios, confidence intervals, and p-values were derived from univariate conditional logistic regression fits for case status*.

Statistical significance is marked with ^*^ for P < 0.05, ^***^ for P < 0.001.

### Gene-Environment Interactions Associated With PSP

Two gene-environment interactions were significantly associated with PSP; however these interactions did not remain significant after FDR correction. Interactions between minor allele of EIF2AK3 and direct chemical exposure, as well as minor allele of EIF2AK3 and years of metal exposure were associated with increased odds for PSP. Participants who carried the minor allele of EIF2AK3 (T) and had direct chemical exposure had almost two times increased odds of PSP compared to those who did not have the minor allele and direct chemical exposure (OR = 1.91, *P* = 0.023, *P*_*FDR*−*corrected*_ =0.71). For every year of metal exposure in individuals with the minor allele of EIF2AK3 (T), the odds of PSP increased by 8.5%, compared to those without EIF2AK3 minor allele (OR = 1.85, *P* = 0.049, *P*_*FDR*−*corrected*_ =0.71). No significant gene-environment interactions were found for SNPs in MAPT, STX6, or MOBP.

### Age of Symptom Onset

**Figure 2:**
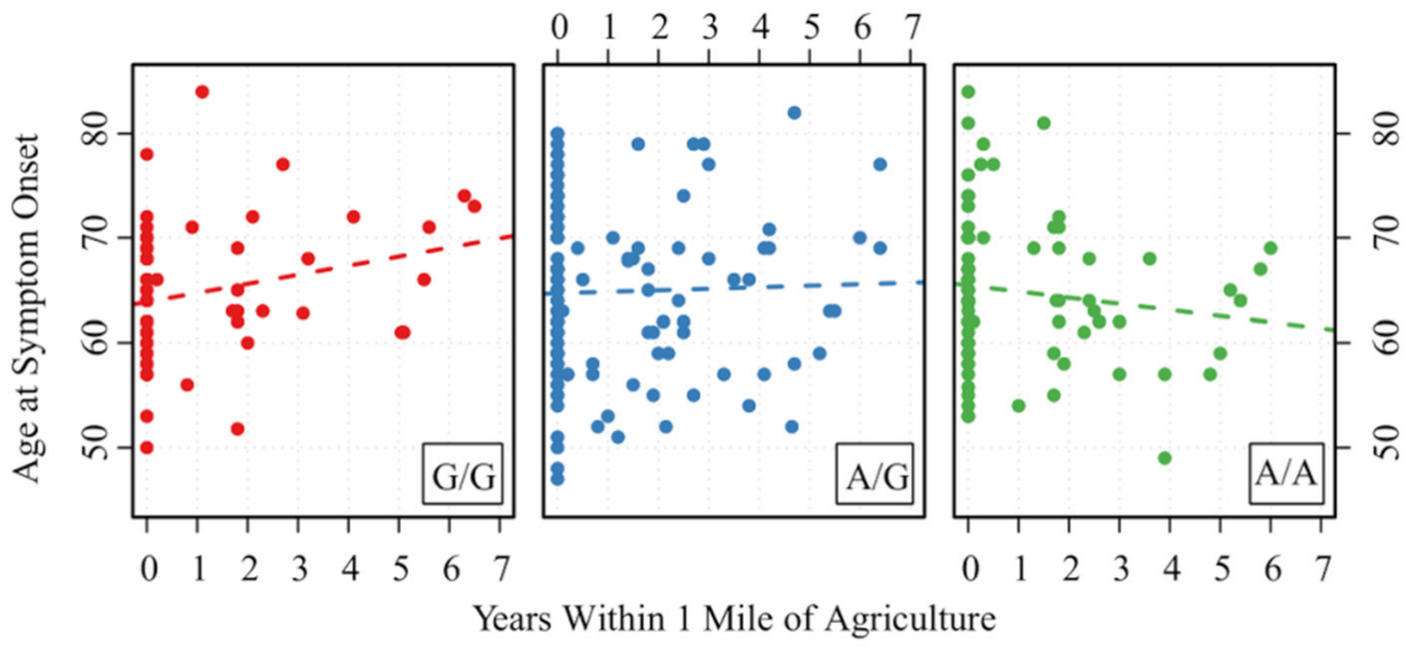
Association between age of PSP symptom onset and the interaction of MAPTrs242557 and years of living within 1 mile of agriculture. A is the minor allele, G is the major allele for MAPTrs242557; G/G corresponds to 0 minor allele, A/G corresponds to 1 minor allele, and A/A corresponds to 2 minor alleles.

**Figure 3:**
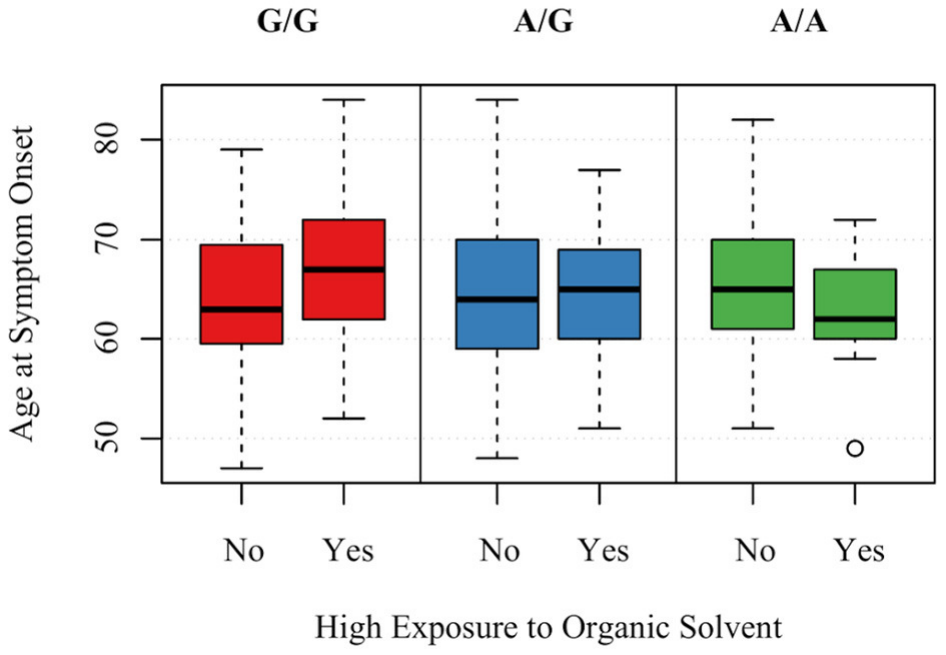
Association between age of PSP symptom onset and the interaction of STX6rs1411478 and exposure to organic solvents. A is the minor allele, G is the major allele for STX6rs1411478; G/G corresponds to 0 minor allele, A/G corresponds to 1 minor allele, A/A corresponds to 2 minor alleles.

Those with the minor allele of MAPTrs8070723 had on older age of symptom onset (*P* = 0.029); for each additional count of minor allele (AA → AG → GG), the mean age of onset increased by 2.825 years. Other SNPs were not associated with age of symptom onset (*P* > 0.89 for all). Two gene-environment interactions were significantly associated with age of onset, although they did not remain significant after FDR correction. For each increased count of MAPTrs242557 minor allele (GG → AG → AA) and 1 year of living within 1 mile of agriculture, the age of onset decreased by 0.724 (*P* = 0.033, *P*_*FDR*−*corrected*_ = 0.68) (Figure 2). For each increased count of STX6rs1411478 minor allele (GG → AG → AA) and high levels of organic solvent, the age of onset decreased by 2.889 (*P* = 0.042, *P*_*FDR*−*corrected*_ =0.68) (Figure 3).

## Discussion

The ENGENE-PSP study used a case-control design to explore the role of gene-environment interactions in the development of PSP and provide preliminary findings for future studies. We hypothesized that sources of oxidative stress, when added to the genetic susceptibility of the MAPT H1 haplotype, may contribute to the development of PSP. Additionally, we hypothesized that sources of endoplasmic reticulum (ER) stress such as cigarette smoke and pesticides might, in conjunction with the EIF2AK3 genetic predisposition, contribute to PSP pathogenesis. We also assessed whether the associations between MOBP, STX6, and PSP would strengthen when combined with an environmental variable.

We found that minor alleles at MAPTrs242557 and EIF2AK3rs7571971 were individually associated with increased odds of developing PSP, and minor alleles of MAPTrs8070723 showed association with lower odds of developing PSP and an older age of symptom onset. The MAPT association with PSP has been long documented (15–17), but this study demonstrated that the association extends beyond the presence or absence of disease, including age of symptom onset. Additionally, prior studies have implicated EIF2AK3 in PSP, but with much larger sample sizes (17); it is interesting that this association remained strong in this study despite the smaller sample size.

We found several potential gene-environment interactions that will require further analysis, given they did not survive FDR-corrections. In unadjusted analyses, combinations of the minor allele of EIF2AK3 and direct chemical exposure, as well as minor allele of EIF2AK3 and years of metal exposure were associated with an increased risk for PSP compared to those without minor alleles of EIF2AK3 and the mentioned exposures. Additionally, the combination of MAPTrs242557 minor allele and years of living within 1 mile of agriculture, as well as STX6rs1411478 minor allele and high levels of organic solvent were associated with an earlier age of symptom onset compared to those without these genetic factors and environmental/occupational exposures.

EIF2AK3 is a gene that encodes PERK, an integral component of the ER unfolded protein response (UPR) (23). Various environmental factors can induce ER stress and activate the UPR, including formaldehyde (a substance in cigarette smoke), alcohol, and paraquat (an ingredient in herbicides) (24). Caparros-Lefebvre et al., reported a cluster of PSP patients in a city with severe environmental contamination by industrial metals in the north of France (14). Therefore, it is not unexpected to find an association between this gene, chemical and metal exposure, and the development of PSP. Finding synergy between genes and environment shown by the association between STX6 and organic solvents for age at symptom onset, despite lack of association between organic solvents and PSP in our previous study in the ENGENE-PSP sample (10) is particularly interesting. However, given the large number of associations tested, we cautiously interpret these associations, but deem them worthy of further investigation in future studies.

The unfavorable effect of combining a MAPT allele with years living within 1 mile of agriculture fits well into the larger narrative of PSP research on etiopathogenesis. MAPT has been well-documented as a significant predisposing gene in PSP, and oxidative stress has been shown to cause tau accumulation (25). Research has implicated various aspects of agriculture as environmental risk factors for PSP development, and though this study did not directly find an association between MAPT and exposure to any specific agents, this combined analysis of MAPT and living within a mile of agriculture does lend further support to a possible interaction (10).

Despite this study being the first to suggest possible gene-environment interactions in PSP, most of these associations did not achieve significance using multivariate analysis. This likely reflects the limited sample size of the study population; although this is the largest case-control study of PSP conducted to date, larger cohorts will be needed to fully examine these interactions. We included five SNPs previously reported to increase the PSP risk in our analysis, and other SNPs reported in PSP (18, 26) should be explored in future studies. Additionally, true gene-environment interactions are likely due to more specific exposures, which we were unable to delineate in this study (i.e., a particular herbicide rather than herbicides in general). Amount of exposure to risk factors also needs to be taken into account as PSP risk can be dose-dependent. Future studies should consider quantification of exposure in association with disease risk. Race is an important factor in genetic studies, as different allele frequencies are observed across different ethnicities (27) and the same SNP may not have the same impact in different races (28). Although we attempted to control for the confounding impact of race in our sample and found the results unchanged, the majority of our sample consisted of White or European-Americans (95.9%) and future studies with more diverse samples are required to determine the race impact for risk factors in PSP. Given that majority of PSP cases had PSP-Richardson's syndrome and a very low number of cases had PSP-parkinsonism, we were unable to assess the gene-environment associations with specific phenotypes in PSP. This is an important point to assess in the future as a GWAS study has suggested TRIM11 locus as a genetic modifier of PSP phenotype (29). Although there is still a need to determine the clinical utility of biomarkers for PSP, future studies would also benefit from incorporating neuroimaging, biofluid and electrophysiological biomarkers [e.g., PET with tau-specific ligands, cerebrospinal fluid tau RT-QuIC (30, 31)] to provide more insight to gene-environment interactions and to strengthen the findings in clinically-diagnosed patients.

In terms of data collection, excluded cases had a significantly shorter disease duration than the included cohort, which may have affected disease severity. For our analysis focusing on gene-environment interactions, we matched cases and controls for race to account for the differences in environmental exposures across different racial groups in the U.S (32). The excluded cases also differed from those included in this study in terms of education and race. The significantly increased proportion of minorities in the excluded cases reflected the challenges of matching such patients with controls for race. These differences in education and race between the included and excluded cases may affect the overall generalizability of the study. Since nearly 10% of the controls in this study were drawn from the spouses or care partners of other participants, this could have artificially masked environmental differences that may have been seen if controls were truly chosen at random. Age of symptom onset was determined based on patient and family report, which may have led to an unreliable estimation of onset as subtle or non-specific symptoms associated with PSP may have been unnoticed before impacting daily life activities significantly. However, the strengths of this study lie in its overall large sample size, careful matching of controls by age, sex, and race, and thorough assessment of several complex genes and environmental exposures that could contribute to PSP pathogenesis.

This case-control, multicenter study is the largest to date to assess gene-environment interactions in PSP and will pave the way for future research into these complex associations. Our explorative study indicates potential interactions between EIF2AK3rs7571971 minor allele and direct chemical exposure, and EIF2AK3rs7571971 minor allele and years of metal exposure for PSP odds, and between MAPTrs242557 minor allele and years of living within 1 mile of agriculture, and STX6rs1411478 minor allele and high levels of organic solvent for age of symptom onset. Studies of larger cohorts are needed to confirm these results and to identify additional interactions. Understanding the specific gene-environment interactions that lead to the development of PSP is integral for our overall understanding of this disease, and for future therapeutic and preventive interventions.

## Data Availability Statement

The raw data supporting the conclusions of this article are available from the corresponding author upon reasonable request.

## Ethics Statement

The studies involving human participants were reviewed and approved by Institutional Review Boards of each participating institution (IRB #111729). The patients/participants provided their written informed consent to participate in this study.

## Author Contributions

IL: conceptualization, obtaining the funding, data collection, and drafting the manuscript. EM, DSt, DR, DH, CM, RD, YB, SR, DSh, BK, CC, GS, and JJ: collection of data. JP and EB: data analysis, and drafting the manuscript. All authors contributed to the interpretation of data and critical review of the manuscript.

## Conflict of Interest

IL supported by the National Institutes of Health grants: 2R01AG038791-06A, U01NS090259, U01NS100610, U01NS80818, R25NS098999, P20GM109025; U19 AG063911-1; 1R21NS114764-01A1; Michael J Fox Foundation, Lewy Body Association, Abbvie, Biogen, Centogene, Roche, EIP-Pharma and Biohaven Pharmaceuticals. She was member of a Lundbeck Advisory Board. She receives her salary from the University of California San Diego and as Chief Editor of Frontiers in Neurology. DSt supported by the Abbvie, Inc., the American Parkinson Disease Association, the Michael J. Fox Foundation for Parkinson Research, Alabama Department of Commerce, the Department of Defense, and NIH grants P50NS108675, R25NS079188, and T32NS095775. He has a clinical practice and is compensated for these activities through the University of Alabama Health Services Foundation. He has served as a consultant for or received honoraria from Abbvie Inc., Sutter Health, the International Parkinson Disease and Movement Disorder Society, Theravance, McGraw Hill, and Sanofi-Aventis. He is a member of the faculty of the University of Alabama at Birmingham and is supported by endowment and University funds. CM received research funding from The Michael J Fox Foundation, Canadian Institutes of Health Research, Parkinson's Foundation (US), International Parkinson and Movement Disorders Society. She is employed by University Health Network, contracted by Grey Matter Technologies, and receives financial compensation as a steering committee member from the Michael J Fox Foundation. DSh employed by Banner Health, received research support from the Arizona Alzheimer's Consortium, Abbvie, Acadia, Aptinyx, Axovant, Biogen, Eisai, Eli Lilly, Enterin, Neurocrine, Michael J Fox Foundation, NIH, Nuvelution, Theravance and Teva; consultant fees from Amneal, Forensis and Neurocrine; speaker honoraria from Acorda, Neurocrine, Sunovion, Teva and US World Meds. The remaining authors declare that the research was conducted in the absence of any commercial or financial relationships that could be construed as a potential conflict of interest.
